# Communication across the bacterial cell envelope depends on the size of the periplasm

**DOI:** 10.1371/journal.pbio.2004303

**Published:** 2017-12-19

**Authors:** Abir T. Asmar, Josie L. Ferreira, Eli J. Cohen, Seung-Hyun Cho, Morgan Beeby, Kelly T. Hughes, Jean-François Collet

**Affiliations:** 1 de Duve Institute, Université catholique de Louvain, Brussels, Belgium; 2 Department of Life Sciences, Imperial College London, London, United Kingdom; 3 Department of Biology, University of Utah, Salt Lake City, Utah, United States of America; 4 WELBIO, Université catholique de Louvain, Brussels, Belgium; Princeton University, United States of America

## Abstract

The cell envelope of gram-negative bacteria, a structure comprising an outer (OM) and an inner (IM) membrane, is essential for life. The OM and the IM are separated by the periplasm, a compartment that contains the peptidoglycan. The OM is tethered to the peptidoglycan via the lipoprotein, Lpp. However, the importance of the envelope’s multilayered architecture remains unknown. Here, when we removed physical coupling between the OM and the peptidoglycan, cells lost the ability to sense defects in envelope integrity. Further experiments revealed that the critical parameter for the transmission of stress signals from the envelope to the cytoplasm, where cellular behaviour is controlled, is the IM-to-OM distance. Augmenting this distance by increasing the length of the lipoprotein Lpp destroyed signalling, whereas simultaneously increasing the length of the stress-sensing lipoprotein RcsF restored signalling. Our results demonstrate the physiological importance of the size of the periplasm. They also reveal that strict control over the IM-to-OM distance is required for effective envelope surveillance and protection, suggesting that cellular architecture and the structure of transenvelope protein complexes have been evolutionarily co-optimised for correct function. Similar strategies are likely at play in cellular compartments surrounded by 2 concentric membranes, such as chloroplasts and mitochondria.

## Author summary

Antibiotic resistance is one of the greatest threats that humanity faces today. In particular, the emergence of multidrug-resistant gram-negative bacteria has become a pressing issue. Infections caused by resistant gram-negative bacteria are indeed difficult to treat because of the presence of a double-membraned envelope that renders these bacteria less permeable to many antibiotics. Bacteria invest a great deal in protecting their envelope. They have evolved complex stress-sensing systems that allow them to monitor envelope integrity in order to detect and fix damage when it occurs. Here, using the model bacterium *Escherichia coli*, we show that the ability to sense and respond to stress depends on the architecture of the cell envelope, in general, and on the distance between the 2 membranes, in particular. Thus, searching for drugs targeting envelope architecture is an attractive strategy for new antibacterials. In addition, our work reveals the physiological function of the Braun’s lipoprotein (Lpp), the numerically most abundant protein in *E*. *coli* and the first lipoprotein that was discovered several decades ago.

## Introduction

Although the multilayered architecture of the cell envelope of gram-negative bacteria was first described in the 1960s, we are still unraveling the links between the structure of this cellular component and its functions in the cell. The envelope of these bacteria consists of an inner membrane (IM), a classical phospholipid bilayer around the cytoplasm, and an outer membrane (OM), an asymmetric structure with phospholipids in the inner leaflet and lipopolysaccharides in the outer leaflet [1]. The space between the IM and the OM defines the periplasm, a cellular compartment that contains the peptidoglycan, a polymer of glycan strands cross-linked by short peptides that provides shape and osmotic protection to cells. This multilayered envelope, which contributes to cellular integrity and modulates permeability, is required for life and serves as an interface to the external milieu. Several essential protein machineries are present in the cell envelope, where they engage in processes that are important for envelope assembly and protection [2–5]. Many of these machineries span the periplasm, with components in both the IM and OM. The pathways that assemble the envelope and that monitor envelope integrity are tightly coordinated in order to sense and respond to damage. However, while the mechanisms of these pathways and how they cooperate to ensure envelope homeostasis have been the focus of numerous studies, the extent to which they depend on the architecture of this compartment for correct functioning is unknown.

To close this gap, here we investigated a key player in the organization of the cell envelope of Enterobacteriaceae: the small, alpha-helical protein Lpp, also known as Braun’s lipoprotein [6]. Lpp is numerically the most abundant protein in *Escherichia coli*, with >1 million copies per cell [7]. Such an extreme abundance suggests that it plays a major role in the cell. Importantly, Lpp provides the only covalent connection between the OM and the peptidoglycan: it is anchored to the OM via a lipid moiety at its N-terminus and attached to the peptidoglycan via its C-terminus. The attachment of Lpp to the peptidoglycan is catalyzed by a family of L,D-transpeptidases [8] that link the C-terminal lysine of Lpp to a diaminopimelic acid residue in the peptide stems of the peptidoglycan [9]. Three *E*. *coli* periplasmic enzymes (YbiS, ErfK, YcfS) exhibit this activity [8]. Both this functional redundancy and the high abundance of Lpp suggest that it must be critically important to covalently connect the OM to the peptidoglycan. However, *lpp* deletion mutants grow and divide normally in culture [10]. The physiological importance of Lpp and of covalently anchoring the OM to the peptidoglycan therefore remains enigmatic.

To control the critical biogenesis of the envelope and maintain its integrity, bacteria have evolved intricate signal transduction systems that induce stress responses to deal with potential dysfunctions and environmental insults to the envelope. In Enterobacteriaceae, the Regulation of Capsule Synthesis (Rcs) system monitors the integrity of the OM and the peptidoglycan [11–13] (S1 Fig). Drugs that interfere with peptidoglycan assembly or alter the lipopolysaccharide leaflet of the OM induce Rcs, which represses cellular motility and produces an extracellular capsule that functions like a protective shield [14]. Rcs is a complex stress-signaling cascade involving at least 6 components [12] (S1 Fig). Most Rcs-inducing cues are sensed by RcsF, an OM-localized lipoprotein, which detects damage caused by chemicals targeting the OM or the peptidoglycan [15, 16] or by mutations in genes involved in envelope assembly [12, 17]. Under stress, RcsF interacts with IgaA, an IM protein, thus constituting a molecular signal that turns on the Rcs pathway via a phosphorylation cascade (S1 Fig) [11, 12]. Rcs therefore mediates signal transduction from the outmost cell layer (the peptidoglycan and the OM) to the control center of the cell (the cytoplasm). To what extent the functioning of a system such as Rcs, with components on both sides of the periplasm, depends on the architecture of the cell envelope remains unknown.

Here, we established that the transmission of stress signals from the OM to the IM depends on the size of the periplasm. By manipulating the length of the lipoprotein Lpp, we demonstrated that cells in which the IM-to-OM distance is artificially increased become blind to stress affecting the peptidoglycan and the OM. Remarkably, we restored the line of communication between the 2 membranes in cells with a larger envelope by increasing the length of the stress sensor lipoprotein RcsF. We also established that the OM must be covalently attached to the peptidoglycan for intermembrane communication to occur.

## Results

**Fig 1:**
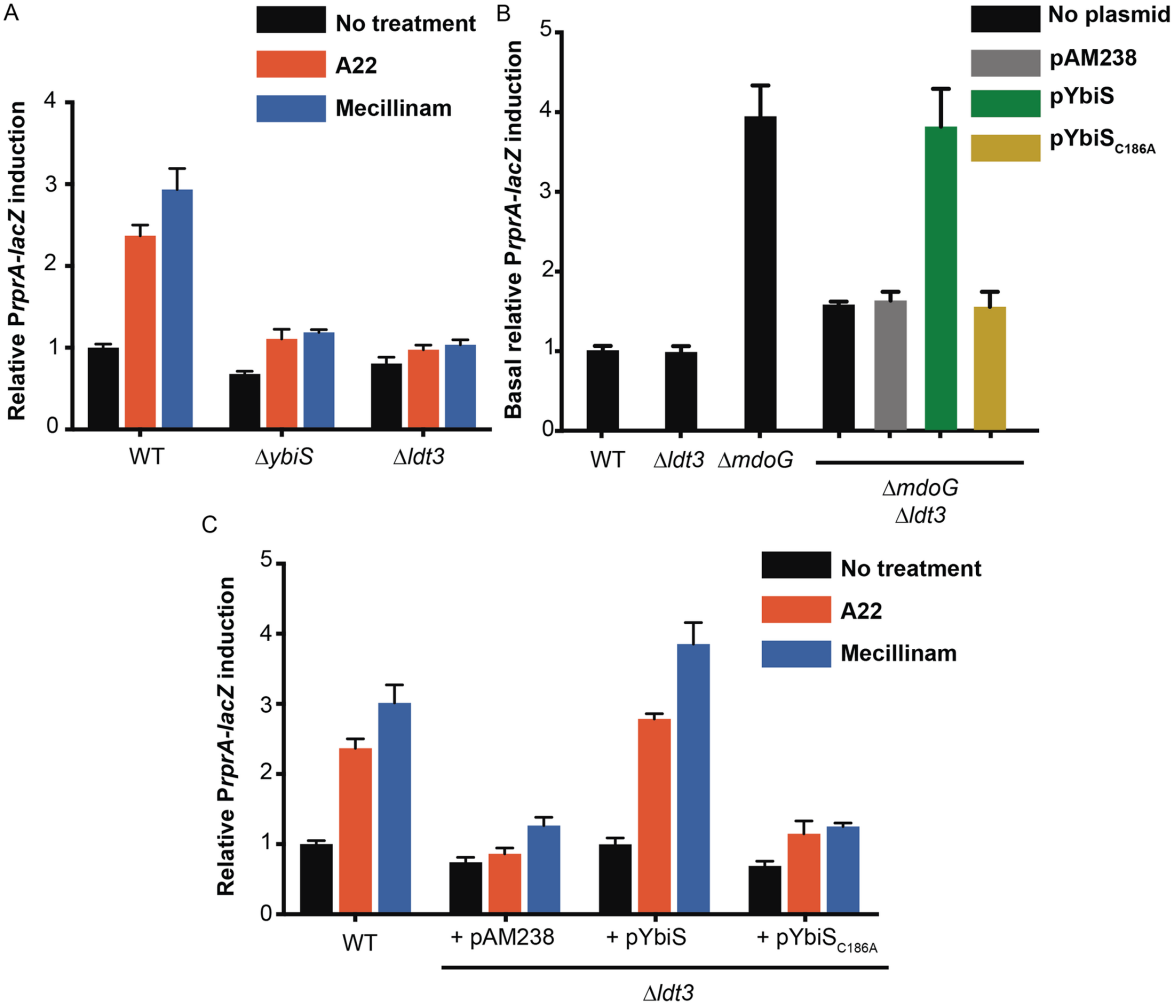
The lack of Lpp-peptidoglycan attachment impairs Rcs activation in response to stress. A chromosomal P*rprA-lacZ* fusion was used to monitor Rcs activity. (A) Deletion of *ybiS* alone (Δ*ybiS*) or of the 3 L,D-transpeptidases (YbiS, ErfK, YcfS) encoded by *Escherichia coli* (Δ*ldt3*) impaired Rcs activity in response to 5 μg/ml A22 or 0.3 μg/ml mecillinam, compared to WT (*F*_(9, 36)_ = 39.45, *P* < 0.0001, 2-way ANOVA). No significant difference was observed between nontreated and treated cells from the mutants. (B) Deletion of *mdoG* constitutively activated Rcs in the WT background (Δ*mdoG*) but not in cells lacking L,D-transpeptidase activity (Δ*ldt3*Δ*mdoG*). Baseline Rcs activity in Δ*mdoG* cells is significantly higher than that in Δ*ldt3*Δ*mdoG* cells (*F*_(6, 14)_ = 68.25, *P* < 0.0001, 1-way ANOVA). Expressing WT YbiS from a low-copy plasmid (pAM238), but not an inactive mutant (YbiS_C186A_), restored Rcs activity in the Δ*ldt3*Δ*mdoG* mutant. Rcs activity in Δ*ldt3*Δ*mdoG* cells harbouring pYbiS was significantly higher than that in Δ*ldt3*Δ*mdoG* cells with empty plasmid or pYbiS_C186A_ (*F*_(6, 14)_ = 68.25, *P* < 0.0001, 1-way ANOVA). (C) Expressing WT YbiS from a low-copy plasmid (pAM238), but not an inactive mutant (YbiS_C186A_), also restored Rcs activity in Δ*ldt3* cells in response to mecillinam and A22. Rcs activity in treated cells harbouring pYbiS was significantly higher than that in treated cells with empty plasmid or pYbiS_C186A_ (*F*_(8, 57)_ = 20.75, *P* < 0.0001, 2-way ANOVA). All values were normalised by the average β-galactosidase activity of untreated WT cells. Error bars depict standard error of the mean (*n* = 6). Lpp, Braun’s lipoprotein; Rcs, regulation of capsule synthesis; WT, wild-type.

In order to interrogate the importance of attaching the OM to the peptidoglycan for the communication of information about cellular stress to the cytoplasm, we triggered the Rcs system in an RcsF-dependent manner [11, 16] with the compound A22, which inhibits the actin-like protein MreB^15^, and mecillinam, a ß-lactam antibiotic, which inhibits the essential transpeptidase PBP2 [18, 19]. Both drugs cause cells to round and eventually lyse [20, 21]. Under these conditions, relative to wild-type (WT) cells, the Rcs pathway was impaired in a mutant lacking YbiS, the primary L,D-transpeptidase [8], and even more in a mutant lacking all 3 L,D-transpeptidases [8] (Δ*ybiS*Δ*erfK*Δ*ycfS* cells, denoted Δ*ldt3* here) (Fig 1A). Note that Δ*ldt3* cells (S2A and S2B Fig) remained susceptible to A22 and mecillinam, becoming round, as expected.

The absence of YbiS, ErfK, and YcfS also impaired Rcs signalling in cells lacking *mdoG* (Fig 1B), a gene involved in the synthesis of periplasmic osmoprotectant sugars. Deletion of *mdoG* has been reported to constitutively activate Rcs via RcsF [22]. Expression of the L,D-transpeptidase YbiS, but not of a catalytically inactive variant, from a plasmid restored Rcs signalling both in the Δ*ldt3*Δ*mdoG* mutant and in the Δ*ldt3* mutant in response to A22/mecillinam (Fig 1B and 1C). Thus, the inability to induce Rcs directly results from the lack of L,D-transpeptidase activity in these cells. Cells expressing an Lpp variant lacking the residue (K58 in *E*. *coli*) required for attachment of the protein to the peptidoglycan (Lpp_ΔK58_) were also unable to trigger Rcs in response to mecillinam, A22, or *mdoG* deletion (S3A and S3B Fig). Taken together, these data indicate that cells become blind to insults that normally activate the Rcs system via RcsF when Lpp is detached from the peptidoglycan.

We then probed whether the failure to induce Rcs resulted specifically from the inability of RcsF to sense stress in cells with no peptidoglycan-linked Lpp. RcsF forms a complex with BamA, the key component of the β-barrel assembly machinery [23], and with the β-barrel proteins OmpA, OmpC, and OmpF [11, 24]. When in complex with these proteins, RcsF is sequestered from IgaA, its downstream partner, and cannot activate Rcs [11]. We previously reported that Rcs-inducing compounds such as A22 and mecillinam decrease the levels of the BamA-RcsF complex [11], likely because newly synthesised RcsF molecules cannot bind BamA under stress. In our model, failure to bind BamA prevents the sequestration of RcsF by β-barrel proteins, allowing RcsF to interact with IgaA and to turn on Rcs [11]. Here, we determined that A22 and mecillinam decreased the levels of BamA-RcsF in Δ*ldt3* cells, similar to the decrease in WT cells (S4A and S4B Fig). Thus, the absence of Rcs induction in cells with no peptidoglycan-bound Lpp (Fig 1, S3A and S3B Fig) did not result from an impaired ability of RcsF to sense stress.

**Fig 2:**
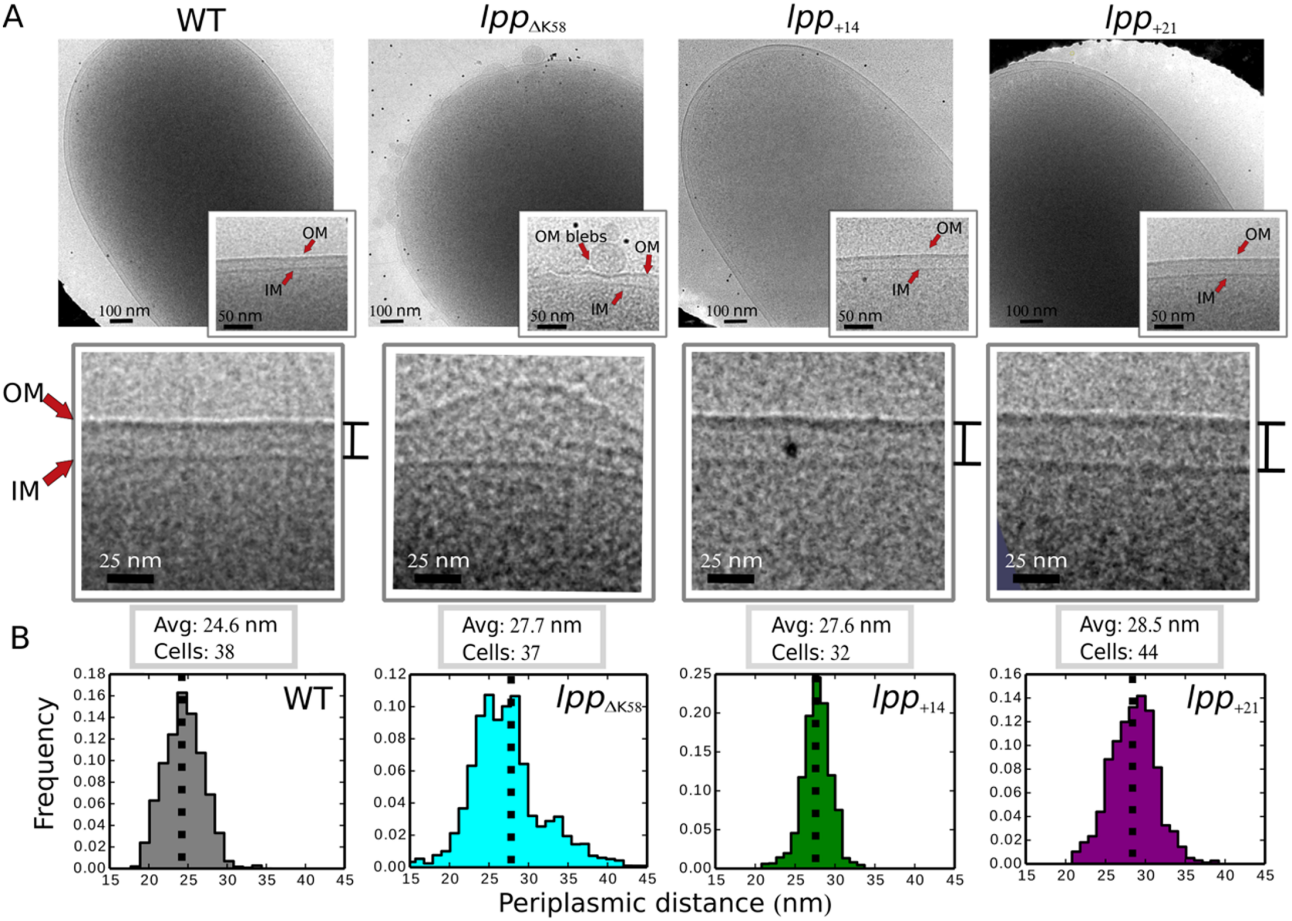
Lpp dictates the distance between the IM and the OM in *Escherichia coli*. (A) Cryo-EM revealed uniform membrane structures with WT cells, but *lpp*_ΔK58_ cells, in which Lpp can no longer bind peptidoglycan, displayed blebbing of the OM. *E*. *coli* strains expressing longer Lpp variants (*lpp*_+14_ and *lpp*_+21_) did not bleb or exhibit membrane defects, similar to WT (S5 Fig). (B) From cryo-EM projection images, distances between the IM and the OM were measured along the cell axis (while avoiding substantial blebbing regions in cells expressing Lpp_ΔK58_) and plotted in 1-nm bins. The *lpp*_ΔK58_ mutant strain had a periplasmic (IM-to-OM) distance that was about 3 nm larger than that of the WT strain (*P* < 0.0001, Kruskal-Wallis) and had a much broader spread of data. The *lpp*_+14_ and *lpp*_+21_ mutant strains had periplasmic distances that were 3 nm and 4 nm, respectively, larger than WT (*P* < 0.0001, Kruskal-Wallis), indicating that the IM-to-OM distance varies as a function of the length of Lpp. Avg, average; cryo-EM, cryo-electron microscopy; IM, inner membrane; Lpp, Braun’s lipoprotein; OM, outer membrane; WT, wild-type.

Recent studies in *Salmonella* concluded that Lpp was a principal determinant of the size of the periplasm [25], prompting us to consider the possibility that in cells with no peptidoglycan-bound Lpp, the structure of the cell envelope is affected in such a way that the OM lipoprotein RcsF cannot reach across the periplasm to contact IgaA in the IM and activate the Rcs system (S1 Fig). Here, cryo-electron microscopy (cryo-EM), a technique in which cells are preserved in a frozen-hydrated, near-native state, revealed that cells expressing Lpp_ΔK58_ formed OM blebs and had envelope defects (Fig 2A). Similar morphological deformities were observed with the *lpp* deletion mutant [25, 26], indicating that, in the case of this mutant, deformities specifically resulted from the absence of covalent anchoring of the OM to the peptidoglycan. Interestingly, the average IM-to-OM distance increased by about 3 nm compared to WT cells (Fig 2B), which suggested that the absence of Rcs induction in cells with no peptidoglycan-bound Lpp could result from a larger periplasm. However, the IM-to-OM distance varied considerably along the cell axis, which complicated further interpretation of the results obtained in cells expressing Lpp_ΔK58_.

Increasing the length of Lpp was previously reported to change the IM-to-OM distance in *Salmonella* without causing OM blebbing [25]. To clearly establish the importance of IM-to-OM distance in Rcs signalling, we engineered *E*. *coli* strains expressing longer Lpp variants (insertions of 14 residues (Lpp_+14_) or 21 residues (Lpp_+21_)) from the native *lpp* locus (S5A Fig); with these strains, we sought to increase the IM-to-OM distance without altering Lpp cross-linking to the peptidoglycan (S5B Fig). Strikingly, cryo-EM revealed that the IM-to-OM distance in these strains increased proportionally to the length of Lpp, with increases of 3 nm and 4 nm in cells expressing Lpp_+14_ and Lpp_+21_, respectively (Fig 2A and 2B).

**Fig 3:**
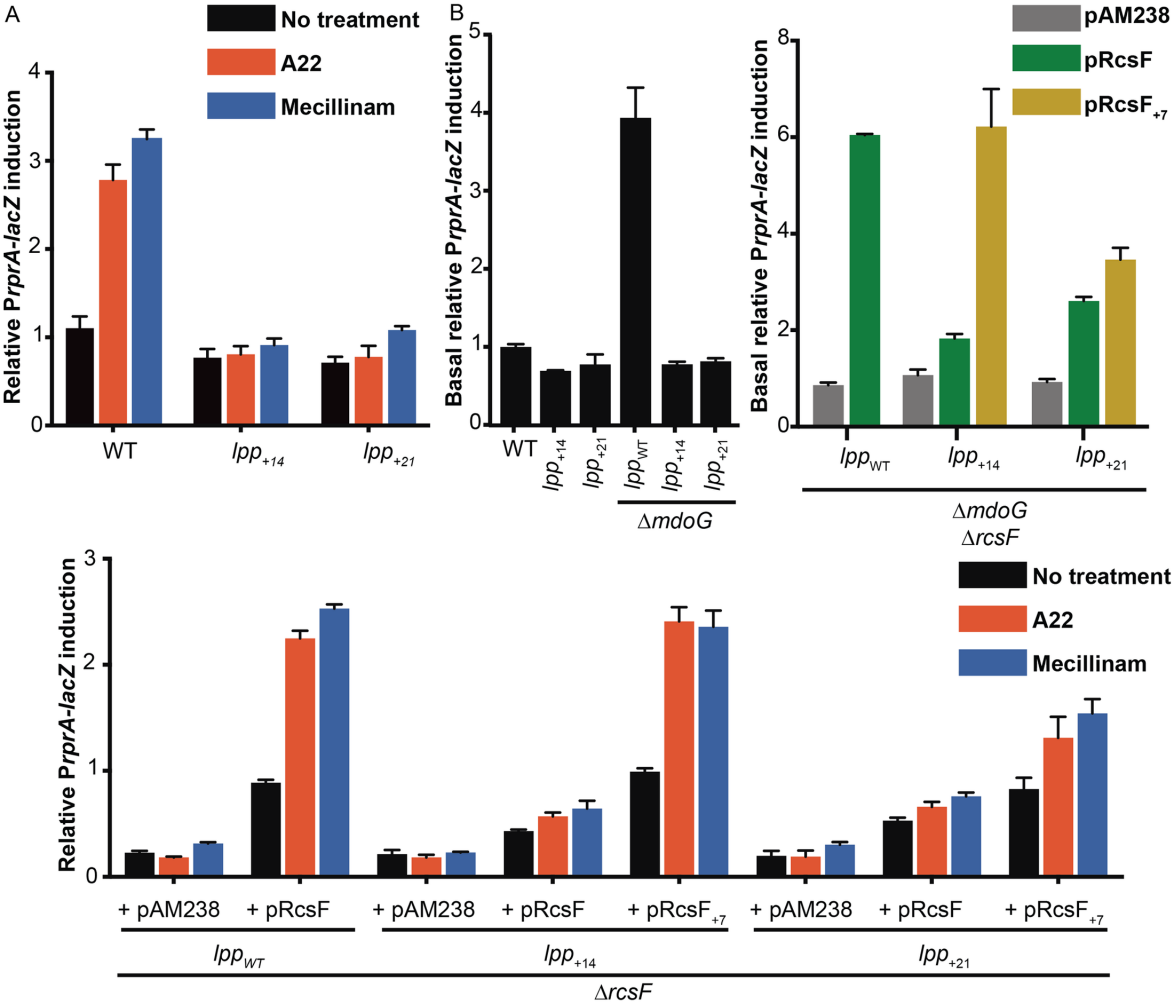
Increasing the length of Lpp impairs Rcs activity during stress, an effect that can be counteracted by extending the N-terminal linker of RcsF. β-galactosidase activity was used as a reporter of Rcs activity as in Fig 1. (A) The lengthened Lpp variants Lpp_+14_ and Lpp_+21_ impaired the activity of the Rcs system in cells treated with 5 μg/ml A22 or 0.3 μg/ml mecillinam compared to WT (*F*_(4, 33)_ = 23.75, *P* < 0.0001, 2-way ANOVA). No significant difference was observed between nontreated and treated mutant cells. (B) Lpp_+14_ and Lpp_+21_ also impaired the Rcs response to *mdoG* deletion compared to WT (*F*_(5, 32)_ = 62.23, *P* < 0.0001, 1-way ANOVA). (C, D) Expressing the extended RcsF mutant (RcsF_+7_) from a low-copy plasmid (pAM238) restored (C) the Rcs response to *mdoG* deletion and (D) the Rcs response to A22 or mecillinam completely or partially in cells expressing Lpp_+14_ or Lpp_+21_, respectively. Rcs activity in Δ*mdoG*Δ*rcsF* cells harbouring pRcsF_+7_ was significantly higher than that in Δ*mdoG*Δ*rcsF* cells with empty plasmid or pRcsF (*F*_(7, 16)_ = 54.94, *P* < 0.0001, 1-way ANOVA). Rcs activity in A22- or mecillinam-treated cells harbouring pRcsF_+7_ was significantly higher than treated cells carrying empty plasmid (pAM238) or pRcsF (*F*_(14, 61)_ = 22.56, *P* < 0.0001, 2-way ANOVA). All values were normalised by the average β-galactosidase activity of untreated WT cells. Error bars represent standard error of the mean (*n* = 6). Lpp, Braun’s lipoprotein; Rcs, regulation of capsule synthesis; WT, wild-type.

We next used the engineered strains to test the impact of increasing the IM-to-OM distance on Rcs signalling. Remarkably, adding 14 or 21 residues to Lpp completely abolished Rcs activation: cells expressing Lpp_+14_ or Lpp_+21_ did not turn on Rcs when exposed to mecillinam or A22 or deleted for *mdoG* (Fig 3A and 3B). Stress was still sensed by RcsF in these strains, as BamA-RcsF levels decreased under stress (S6A and S6B Fig). In addition, retargeting RcsF to the IM by altering its sorting signal constitutively induced Rcs (S7 Fig), as expected [15], indicating that Rcs could still be turned on in strains expressing Lpp_+14_ or Lpp_+21_. Interestingly, increasing the length of Lpp was well tolerated: cells expressing Lpp_+14_ or Lpp_+21_ exhibited no growth defects (S5C Fig), did not bleb (Fig 2A), and had WT morphology except for a larger periplasm (Fig 2A). In addition, expression of Lpp_+14_ or Lpp_+21_ fully complemented the sensitivity of the Δ*lpp* mutant to the membrane perturbant dibucaine [27, 28] (S5D Fig) and complemented the growth defect of the Δ*mrcB*Δ*lpp* double mutant [29] (S5E Fig).

The results above are consistent with the hypothesis that RcsF does not activate the Rcs system when the size of the periplasm increases because RcsF cannot reach IgaA. If this hypothesis is correct, then making RcsF longer should allow this protein to span the increased IM-to-OM distance in order to reach IgaA and activate Rcs. RcsF has a 32-residue unstructured linker that is located upstream of the signalling domain [30, 31] and that most likely allows the protein to reach the IM. To make RcsF longer, we added 7 residues to the C-terminal part of the linker (RcsF_+7_; S8A and S8B Fig). Because this sequence is disordered (S8C and S8D Fig), we predicted that adding it to the linker would increase the length of RcsF by 2–3 nm (S8C Fig), roughly corresponding to the increase in length resulting from the addition of a 14-residue α-helix to Lpp (strain *lpp*_+14_). Remarkably, expression of RcsF_+7_ in cells producing Lpp_+14_ fully restored Rcs signalling in response to *mdoG* deletion, A22, or mecillinam (Fig 3C and 3D). It also partially rescued signalling in cells expressing Lpp_+21_ (Fig 3C and 3D), in which the periplasmic size is larger than WT by 4 nm (Fig 2B). Thus, increasing the length of RcsF restored normal Rcs signalling in cells in which the IM-to-OM distance was increased to a similar extent. These observations support our model that, in cells with a larger IM-to-OM distance, the absence of Rcs induction under stress results from the inability of RcsF to reach the other side of the periplasm (S9 Fig).

## Discussion

Taken together, our results reveal the importance of controlling the IM-to-OM distance in the maintenance of intermembrane communication within the cell envelope, establishing the physiological importance of the size of the periplasm and highlighting the exquisite architectural organisation of the envelope. In particular, we demonstrated that strict control over the IM-to-OM distance is required for effective envelope surveillance and protection during exponential growth. Even a slight increase in the size of the periplasm disrupted the line of communication between the OM and the IM, effectively disconnecting the outer part of the envelope from the cytoplasm, where cellular behaviour is controlled (S9 Fig). Thus, envelope components have likely been subjected to evolutionary pressure to properly function in the strictly controlled dimensions of the periplasm. For example, the sizes of RcsF and of the periplasmic domain of IgaA must have been rigorously selected to allow the optimal transmission of stress signals between these 2 proteins across the envelope.

In addition, our observation that cells expressing longer Lpp variants had no phenotype in the conditions tested (Fig 2A and S5B–S5E Fig) reveals that the essential envelope-spanning machineries involved in elongation and division have enough intrinsic flexibility to adapt to changes in envelope architecture, suggesting that evolution has selected for robustness in the case of these systems. These results motivate future research to test whether the correct functioning of other envelope-spanning systems in *E*. *coli* and other bacteria also depends on strict control over IM-to-OM distance and to determine the evolutionary advantage that makes the Rcs system so susceptible to changes in the size of the periplasm.

Recent work revealed that Lpp functions as an OM tether under normal growth conditions in *Salmonella* [25]. Accordingly, removing the covalent connection between OM-anchored Lpp and the peptidoglycan leads to the formation of OM blebs in *E*. *coli* (Fig 2A). Interestingly, although the average IM-to-OM distance was larger in cells expressing Lpp_ΔK58_ than in cells expressing WT Lpp, WT size was maintained in substantial portions of the envelope (Fig 2B). A likely explanation is that in the absence of peptidoglycan-bound Lpp, envelope proteins such as the lipoprotein Pal and the β-barrel OmpA, which noncovalently bind peptidoglycan [32, 33], partially compensate for the loss of Lpp periplasmic spanners, sustaining WT size in some envelope areas. Strikingly, in cells expressing Lpp_+14_ or Lpp_+21_, the IM-to-OM distance increased homogeneously along the cell axis, and no WT size was observed (Fig 2A and 2B), suggesting that these longer versions of Lpp override proteins like Pal and OmpA and impose a larger periplasm. Thus, Lpp may act as both a tether and a support column for the OM.

Lpp was the first lipoprotein to be identified [6]. However, the functional importance of this massively abundant protein remained poorly defined. Fifty years later, the crucial role of Lpp in controlling the architecture of the bacterial cell envelope, and in particular the IM-to-OM distance, is finally coming to light. The current investigation has revealed the importance of controlling this distance in the maintenance of intermembrane communication within the cell envelope. Cellular compartments surrounded by 2 concentric membranes occur in all kingdoms of life. By highlighting the importance of controlling the intermembrane distance in gram-negative bacteria, our work suggests that similar strategies are at play in other cellular compartments surrounded by 2 concentric membranes, such as chloroplasts and mitochondria.

## Materials and methods

### Bacterial strains and plasmids

Bacterial strains and plasmids used in this study are listed in S1 and S2 Tables, respectively. *E*. *coli* K12 strain MG1655 was used as WT throughout the study. For all deletion mutants, the corresponding alleles from the KEIO collection [34] were transferred into MG1655 or derivatives carrying a chromosomal P*rprA-lacZ* fusion at the phage lambda attachment site [35] via P1 phage transduction and validated with PCR. To excise the kanamycin-resistance cassette (*kanR*), pCP20 was used as described previously [36].

### Construction of *lpp* length mutants

*lpp* length mutants were constructed in *E*. *coli* K12 F^-^ λ^-^ cells harbouring the λRed recombineering plasmid (pKD46) [36], as described previously [25]. Briefly, a tetracycline-resistance cassette (*tetRA*) was inserted between codons 42 and 43 of *E*. *coli lpp*. Replacement of the *tetRA* cassette with insertions of 14 or 21 residues (2 and 3 heptad repeats, respectively) was accomplished by introducing dsDNA fragments bearing 40 bp of homology to *E*. *coli lpp* directly flanking the 5′ and 3′ ends of the *tetRA* cassette. Tetracycline-sensitive transformants (*lpp* length mutants) were selected by plating on medium containing anhydrotetracycline (0.2 mg/ml) and fusaric acid (2.4 mg/ml). Chromosomal *lpp* length mutations were moved into the MG1655 background through standard λRed recombineering as described in [37]. Primer sequences are available upon request.

### Construction of the *lpp*_ΔK58_ mutant

The *lpp*_ΔK58_ mutant was constructed using site-directed mutagenesis of WT *lpp* cloned into pBad18, as previously described [38]. *lpp*_ΔK58_ was moved onto the chromosome of MG1655 cells via standard λRed recombineering, as described in [37].

### Construction of RcsF_+7_

To create a longer version of RcsF, we used pSC202 (the RcsF ORF in the low-copy vector pAM238) [36] as a template (S2 Table). The extended linker fragment was synthesised with flanking restriction sites RsrII and PstI using gene fragments (sequence available upon request). The fragment and vector pSC202 were digested and ligated to yield pRcsF_+7_.

### β-Galactosidase assay

β-galactosidase activity was measured as described previously [39]. Briefly, cells harbouring P*rprA*-*lacZ* at the *att*B phage lambda site on the chromosome were diluted 1:100 from overnight cultures in Luria broth (LB), then incubated at 37°C. When needed, the cultures were treated with 0.3 μg/ml mecillinam or 5 μg/ml A22 at OD_600_ = 0.2. Cells were further grown for 1 h (with mecillinam treatment) or 40 min (with A22 treatment). Otherwise, cells were harvested at OD_600_ = 0.6. Twenty microlitres of cells were harvested and incubated with 80 μl of permeabilisation solution (60 mM Na_2_HPO_4_·2H_2_O, 40 mM NaH_2_PO_4_·H_2_O, 10 mM KCl, 1 mM MgSO_4_·7H_2_O, 50 mM β-mercaptoethanol) for 30–45 min at room temperature. Then, 600 μl of substrate (1 mg/ml O-nitrophenyl-β-d-galactoside, 50 mM β-mercaptoethanol) were added. The mixture was further incubated at 30°C for 20–25 min. Seven hundred microlitres of 1 M Na_2_CO_3_ were added to stop the reaction, and the optical density was measured at 420 nm. The standardized amount of β-galactosidase activity was reported in Miller units. The ratio of P*rprA-lacZ* induction was calculated relative to the basal level in a WT strain. Bar graphs with corresponding statistical analysis were prepared using Prism 7 (GraphPad Software, Inc.).

### In vivo 3,3′-dithiobis[sulfosuccinimidylpropionate] (DTSSP) cross-linking

Cells were grown in LB until OD_600_ = 0.5. One millilitre of cells was harvested via centrifugation at 3,300 × *g* for 5 min at 4°C, washed, and resuspended in phosphate-buffered saline (PBS). The cell suspension was split in half and DTSSP (Thermo Scientific), prepared fresh in PBS, was added to 1 of the 2 samples to a final concentration of 2 mM. The DTSSP-treated and nontreated samples were incubated at 30°C for 1 h under orbital agitation. Glycine (0.1 M) was added to both samples to quench cross-linking. After 10 min on ice, the cell suspensions were precipitated with 10% trichloroacetic acid (TCA), washed with ice-cold acetone, and resuspended in Laemmli SDS sample buffer (2% SDS, 10% glycerol, 60 mM Tris-HCl [pH 7.4], 0.01% bromophenol blue). The volume of sample buffer used to resuspend the samples was normalised to the number of cells loaded. The samples were subjected to SDS-PAGE and immunoblotting using specific antibodies, as described below. Only data from DTSSP-treated samples are shown in S4 and S6 Figs.

### SDS-PAGE and immunoblotting

Protein samples were separated in 12% or 4%–12% acrylamide gels (Life Technologies) and transferred to nitrocellulose or polyvinylidene fluoride membranes (Whatman, 0.45 μm). Immunoblotting was performed as described previously [11]. Rabbit anti-RcsF (from the Collet laboratory’s collection) and rabbit anti-Lpp (from the Hughes laboratory’s collection) were diluted 1:10,000 in 1% skim milk, TBS-T (50 mM Tris-HCl [pH 7.6], 0.15 M NaCl, 0.1% Tween 20). The membranes were incubated with horseradish peroxidase-conjugated goat anti-rabbit IgG (Sigma; 1:10,000 in 1% skim milk, TBS-T) and washed with dilution buffer. Labelled proteins were detected via enhanced chemiluminescence (Pierce ECL Western Blotting Substrate, Thermo Scientific), which was imaged with a GE ImageQuant LAS4000 camera (GE Healthcare Life Sciences). To measure RcsF levels, band intensities were quantified using ImageJ 1.48v (NIH) and analysed with ImageQuant TL software 1Dv8.1 to ensure that they were within the linear range.

### Phase-contrast microscopy

Strains were grown to an OD_600_ of 0.5. Then, 450 μl of culture was collected and mixed with 50 μl of 10× fixing solution (0.4% glutaraldehyde, 25% formaldehyde, 330 mM sodium phosphate [pH 7.6]) for 30 min at room temperature. Cells were spun down at 6,000 × *g* at room temperature, resuspended in 50 μl PBS, and imaged on agarose pads.

Fixed samples were imaged with a Primo Star microscope (Carl Zeiss) equipped with an AxioCam 105 color camera (Carl Zeiss) and a phase-contrast objective (Plan-Achromat 40×/0.65 Ph2; Carl Zeiss). Images were acquired with Zen 2 (blue edition; Carl Zeiss). Exposure times and image scaling were identical for compared conditions. MicrobeTracker [40] was used to obtain cell outlines. Quantitative analysis from cell meshes was done with MATLAB R2014a (Mathworks, Inc.) using custom scripts to plot the distributions of length-to-width ratios for the strains tested.

### Cryo-EM

Strains were grown aerobically in LB at 37°C until an OD_600_ of 0.6 was reached. Cells were spun for 5 min at 6,000 × *g* at 4°C and resuspended to an OD_600_ of about 12.

UltraAuFoil R2/2 grids (200 mesh; Quantifoil Micro Tools GmbH) were glow-discharged for 60 s at 10 mA. Cells were mixed with a solution of 10 nm colloidal gold (Sigma) immediately before freezing. A 2.5-μl droplet of sample was applied to the grid and plunge frozen using a Vitrobot MkIV (FEI Company) with a wait time of 60 s, a blot time of 5 s, a blot force of 3, and a drain time of 1 s at a constant humidity of 100%. Grids were stored under liquid nitrogen until required for data collection.

Projection images were collected on a 200 keV FEI Tecnai TF20 FEG transmission electron microscope (FEI Company) equipped with a Falcon II direct electron detector (FEI Company) using a Gatan 626 cryogenic-holder (Gatan). Leginon automated data-collection software 3.0 [41] was used to acquire images with pixel size of 0.828 nm (nominal magnification 25,000×) with a defocus of −5 μm. Membrane measurements were carried out as previously described [25]. Briefly, 3dmod from the IMOD package [42] and custom scripts were used to manually segment the IMs and OMs of projection images of about 35 cells per mutant, measuring the periplasmic width at 0.5-nm intervals to produce width histograms (Fig 2B).

### Growth analysis

Single colonies were used to inoculate overnight cultures, which were diluted 1:1,000 in round-bottom 96-well plates in 200 μl LB. Absorbance was measured at 600 nm every 30 min in a Synergy H1 microplate reader (BioTek) with constant orbital shaking at 37°C. Graphs were prepared using GraphPad Prism 7.

### Dibucaine sensitivity assay

Sensitivity to the membrane perturbant dibucaine was assessed on LB agar plates. Briefly, 4-ml cultures were inoculated with overnight cultures at 1:100 dilution and grown in LB at 37°C until an OD_600_ of about 0.5 was reached. Cell counts were normalised according to OD_600_, then serially diluted in LB with seven 10-fold dilutions using 96-well microtitre plates (Corning). Two microlitres of the diluted cultures were manually spotted onto the LB agar plates and incubated overnight at 37°C. When indicated, dibucaine (Sigma-Aldrich) was added to the LB agar plate at a final concentration of 1.2 mM.

### Detection of Lpp linked to cell-wall sacculi

Cultures were grown until OD_600_ = 0.7 in LB at 37°C with agitation. Cells (1 ml) were pelleted and resuspended in 1 ml PBS. When indicated, 1 mg lysozyme (Sigma-Aldrich) and EDTA (final concentration 10 mM) were added to the resuspended cells, which were incubated for 1 h at 37°C. Lysates were obtained via TCA precipitation and resuspension in 100 μl 2× SDS Laemmli buffer. Typically, 5–10 μl of sample were loaded into 12% Bis-Tris polyacrylamide gels (Nupage). Lpp was detected using anti-Lpp antiserum.

### Statistical methods

The significance of differences among bacterial strains was assessed using GraphPad Prism 7 according to analysis of variance (ANOVA), followed by the application of Tukey’s multiple-comparison test when the distribution was normal. Otherwise, the Kruskal-Wallis test was used, followed by Dunn’s multiple comparison test. Normality was assessed using the Shapiro-Wilk test.

## Supporting information

S1 Table Strains used in this study. (XLSX)

S2 Table Plasmids used in this study. (XLSX)

S1 Fig The Rcs phosphorelay system detects envelope stress due to alterations to the OM or the peptidoglycan. The OM lipoprotein RcsF senses most of the cues activating the Rcs system. Under stress conditions, RcsF interacts with the inner membrane protein, IgaA, alleviating its inhibitory effect on the Rcs system. How IgaA down-regulates the Rcs system is unknown. When the system becomes activated, the histidine kinase RcsC autophosphorylates, then transfers the phosphate to RcsD and thence to the response regulator RcsB. Phosphorylated RcsB forms homodimers or heterodimers with RcsA to regulate the expression of target genes involved in motility, colanic acid synthesis, biofilm formation, osmotic homeostasis, and periplasmic quality control [13, 35]. OM, outer membrane; Rcs, regulation of capsule synthesis.
(TIF)

S2 Fig Cells lacking Lpp-peptidoglycan cross-links round upon A22 or mecillinam treatment. (A) Representative phase-contrast images reveal that Δ*ldt3* cells rounded upon treatment with A22 (5 μg/ml) or mecillinam (0.3 μg/ml). (B) Length-to-width ratio distributions for cells in (A). There was no significant difference between mean ratios from WT cells and Δ*ldt3* cells. Both presented an overlapping rounding distribution upon A22 or mecillinam treatment with no significant difference (*F*_(2, 1881)_ = 4.599, *P* = 0.6541 for A22, and *P* = 0.9165 for mecillinam, 2-way ANOVA). Lpp, Braun’s lipoprotein; std, standard deviation; WT, wild-type.
(TIF)

S3 Fig The Rcs response to stress is impaired in cells expressing Lpp_ΔK58_. β-galactosidase activity was measured as in Fig 1. (A) Cells harbouring Lpp_ΔK58_ (encoded on the chromosome at the *lpp* locus) were unable to activate the Rcs system in response to 5 μg/ml A22 or 0.3 μg/ml mecillinam, compared to WT (*F*_(2, 27)_ = 14.66, *P* < 0.0001, 2-way ANOVA). (B) The *lpp*_ΔK58_ mutant also displayed an impaired Rcs response to *mdoG* deletion (*F*_(3, 20)_ = 45.86, *P* < 0.0001, 1-way ANOVA). All values were normalised by the average activity for untreated WT cells. Error bars depict standard error of the mean (*n* = 6 for A22 and *n* = 3 for mecillinam). Lpp, Braun’s lipoprotein; Rcs, regulation of capsule synthesis; WT, wild-type.
(TIF)

S4 Fig RcsF still senses stress when Lpp is not attached to the peptidoglycan. (A, B) Immunoblots show that levels of the BamA-RcsF complex were significantly lower after treatment (A) with A22 (5 μg/ml) or (B) with mecillinam (0.3 μg/ml) in WT and mutant strains (*F*_(2, 12)_ = 50.48, *P* < 0.0001, 2-way ANOVA). BamA-RcsF complex levels were normalised to unspecific cross-reacting bands of the RcsF antibody. BamA-RcsF ratios were calculated relative to their levels in the no-stress condition for each strain. Representative data are shown from experiments performed in biological triplicate. Lpp, Braun’s lipoprotein; WT, wild-type.
(TIF)

S5 Fig Cells expressing the Lpp variants Lpp_+14_ and Lpp_+21_ show an overall phenotype that is similar to WT. (A) Linear sequences of *lpp* mutants with insertions of 14 or 21 residues (2 and 3 heptad repeats, respectively). Insertions are underlined. (B) All Lpp variants expressed at similar levels from the chromosome cross-link to peptidoglycan. Lpp-peptidoglycan cross-links are detected in samples treated with lysozyme. Representative data are shown from experiments performed in biological triplicate. (C) Cells expressing Lpp_+14_ or Lpp_+21_ from the chromosome grow similarly to WT and to Δ*lpp* and *lpp*_ΔK58_ mutants at 37°C. Values are averaged from independent clones (*n* = 6); error bars depict standard deviation. (D) Cells expressing Lpp_+14_ or Lpp_+21_ from the chromosome are as resistant as WT to dibucaine, unlike Δ*lpp* and *lpp*_ΔK58_ mutants. Representative data are shown from experiments performed in biological triplicate. (E) Cells bearing *lpp*_+14_ or *lpp*_+21_ on the chromosome in addition to the *mrcB* deletion exhibited no significant growth defect compared to Δ*lpp*Δ*mrcB* mutants. Growth analysis was performed (*n* = 6) and depicted as in panel (C). Lpp, Braun’s lipoprotein; WT, wild-type.
(TIF)

S6 Fig RcsF senses stress in cells expressing Lpp_+14_ and Lpp_+21_. (A, B) Immunoblots indicate that levels of the BamA-RcsF complex were significantly lower after treatment (A) with 5 μg/ml A22 or (B) with 0.3 μg/ml mecillinam in WT and mutant strains (*F*_(2, 18)_ = 58.08, *P* < 0.0001, 2-way ANOVA). Cells were treated with (A) A22 or (B) mecillinam and harvested after 40 min or 1 h, respectively. DTSSP cross-linking was performed, followed by SDS-PAGE and immunoblotting with an anti-RcsF antibody, as in S4 Fig. BamA-RcsF complex levels were normalised to unspecific cross-reacting bands of the RcsF antibody. BamA-RcsF ratios were calculated relative to their levels in the no-stress condition for each strain. Representative data are shown from experiments performed in biological triplicate. DTSSP, 3,3′-dithiobis[sulfosuccinimidylpropionate]; Lpp, Braun’s lipoprotein; SDS-PAGE, Sodium dodecyl sulfate–polyacrylamide gel electrophoresis; WT, wild-type.
(TIF)

S7 Fig Retargeting RcsF to the IM by altering its sorting signal constitutively induces Rcs in cells expressing Lpp_+14_ and Lpp_+21_. β-galactosidase activity was measured as in Fig 1. Expressing the RcsF mutant retargeted to the IM (RcsF_IM_) from a low-copy plasmid (pAM238) constitutively activated the Rcs response in cells expressing Lpp_+14_ and Lpp_+21_, as in cells expressing Lpp_WT_. Activation levels were significantly higher when expressing RcsF_IM_ (*F*_(16, 43)_ = 187.2, *P* < 0.0001, 1-way ANOVA). All values were normalised to the average activity obtained for untreated Δ*rcsF* cells, expressing *lpp*_WT_ and harbouring pRcsF. Error bars represent standard error of the mean (*n* = 3). IM, inner membrane; Lpp, Braun’s lipoprotein; Rcs, regulation of capsule synthesis.
(TIF)

S8 Fig Engineering the RcsF_+7_ variant by adding a disordered segment to RcsF_WT_. (A) The functional domains of RcsF. The signal sequence is followed by the lipobox, which contains the acylated cysteine, the first amino acid of the mature lipoprotein. Amino acids at positions +2 and +3 after the cysteine form the Lol sorting signal that allows RcsF to be exported to the outer membrane. The globular signalling domain is preceded by an unstructured linker of 32 residues. (B) Primary sequences of RcsF_WT_ and RcsF_+7_. Colours correspond to the colours in (A). The linker region (yellow) is rich in disorder-promoting amino acids (proline, alanine, lysine, arginine). To construct RcsF_+7_, 7 disordered residues from the WT linker sequence were duplicated. The duplicated peptide in RcsF_+7_ is underlined in yellow. (C) Ribbon representation of the RcsF_WT_ structure (PDB: 2L8Y). This structure, which was solved via nuclear magnetic resonance, contains a flexible linker in the N-terminal part of RcsF_WT_ [31]. The duplicated residues from RcsF_WT_ used to construct RcsF_+7_ are highlighted in yellow on the ribbon using UCSF Chimera [43]. Peptide length was predicted by PyMOL [44] as 2–3 nm. (D) PrDOS [45] prediction of disordered regions in RcsF_WT_ and RcsF_+7_. RcsF_+7_ shows disorder in its extended linker that is similar to that in the WT linker. The input sequence starts at the acylated cysteine residue. The sequence is predicted to be disordered when the disorder probability/confidence score is >0.5. The orange line shows the confidence of disordered protein-binding residue predictions. False positive rates = 5%. Lol, lipoprotein outer membrane localization; PrDOS, protein disorder prediction server; WT, wild-type.
(DOCX)

S9 Fig The distance between the IM and the OM is critical for efficient envelope surveillance and protection. When the bacterium is subjected to stress in the OM or in the peptidoglycan, it activates the Rcs system to control and fix the damage. (A) In order to activate Rcs, the stress sensor lipoprotein RcsF, which is localised in the OM, must cross the periplasm to reach the IM protein IgaA. IgaA functions as the down-regulator of the Rcs system. When interacting with RcsF, IgaA alleviates its inhibition of Rcs, triggering the Rcs response. The architecture of the cell envelope needs to be tightly controlled to allow the information to be transmitted from RcsF in the OM to IgaA in the IM. In WT cells, the IM-to-OM distance is maintained by Lpp, a trimeric [46] lipoprotein that cross-links the OM to the peptidoglycan. (B) Preventing the attachment of Lpp to the peptidoglycan (Lpp_ΔK58_) or (C) making this lipoprotein longer (Lpp_+14_ and Lpp_+21_) increases the width of the periplasm, which disrupts the line of communication between the 2 membranes. (D) Increasing the length of the RcsF N-terminal linker restores Rcs signalling by allowing RcsF to span the wider cell envelope and contact IgaA. IM, inner membrane; Lpp, Braun’s lipoprotein; OM, outer membrane; Rcs, regulation of capsule synthesis; WT, wild-type.
(TIF)

S1 Data Underlying data for Figs 1A, 1B, 1C, 2B, 3A, 3B, 3C, 3D, S2B, S3A, S3B, S4A, S4B, S5C, S5E, S6A, S6B and S7. (XLSX)

## Abbreviations

Cryo-EM: cryo-electron microscopy
IM: inner membrane
Lpp: Braun’s lipoprotein
OM: outer membrane
Rcs: regulation of capsule synthesis
WT: wild-type
