# Supplementary figures and images for: Communication across the bacterial cell envelope depends on the size of the periplasm
Source: PLoS Biol. 2017 Dec 19;15(12):e2004303. doi: 10.1371/journal.pbio.2004303 (PMC5736177; doi:10.1371/journal.pbio.2004303)

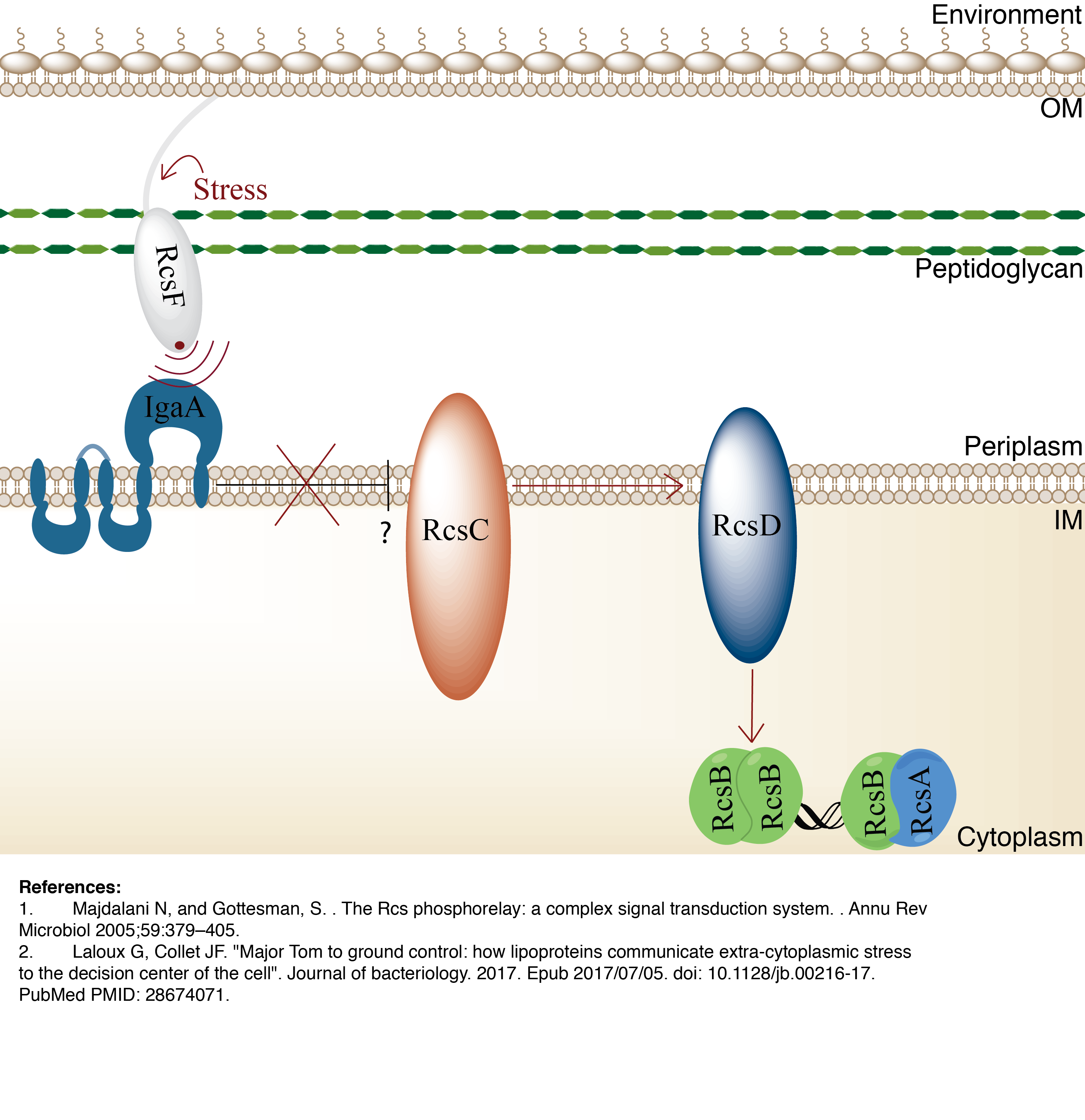

Supplement: S1 Fig — The OM lipoprotein RcsF senses most of the cues activating the Rcs system. Under stress conditions, RcsF interacts with the inner membrane protein, IgaA, alleviating its inhibitory effect on the Rcs system. How IgaA down-regulates the Rcs system is unknown. When the system becomes activated, the histidine kinase RcsC autophosphorylates, then transfers the phosphate to RcsD and thence to the response regulator RcsB. Phosphorylated RcsB forms homodimers or heterodimers with RcsA to regulate the expression of target genes involved in motility, colanic acid synthesis, biofilm formation, osmotic homeostasis, and periplasmic quality control [13, 35]. OM, outer membrane; Rcs, regulation of capsule synthesis. (TIF) [file pbio.2004303.s003.tif]

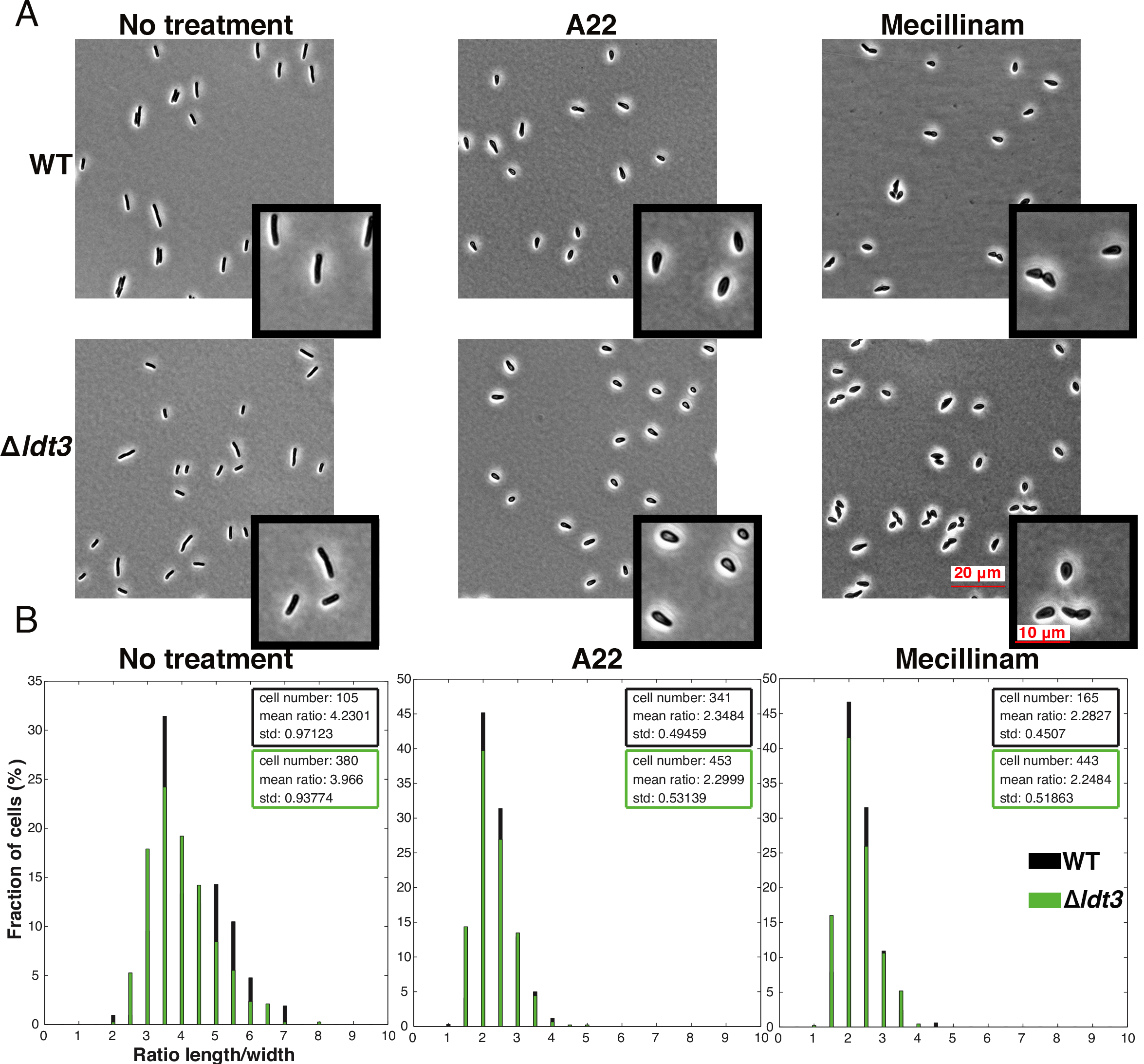

Supplement: S2 Fig — (A) Representative phase-contrast images reveal that Δldt3 cells rounded upon treatment with A22 (5 μg/ml) or mecillinam (0.3 μg/ml). (B) Length-to-width ratio distributions for cells in (A). There was no significant difference between mean ratios from WT cells and Δldt3 cells. Both presented an overlapping rounding distribution upon A22 or mecillinam treatment with no significant difference (F(2, 1881) = 4.599, P = 0.6541 for A22, and P = 0.9165 for mecillinam, 2-way ANOVA). Lpp, Braun’s lipoprotein; std, standard deviation; WT, wild-type. (TIF) [file pbio.2004303.s004.tif]

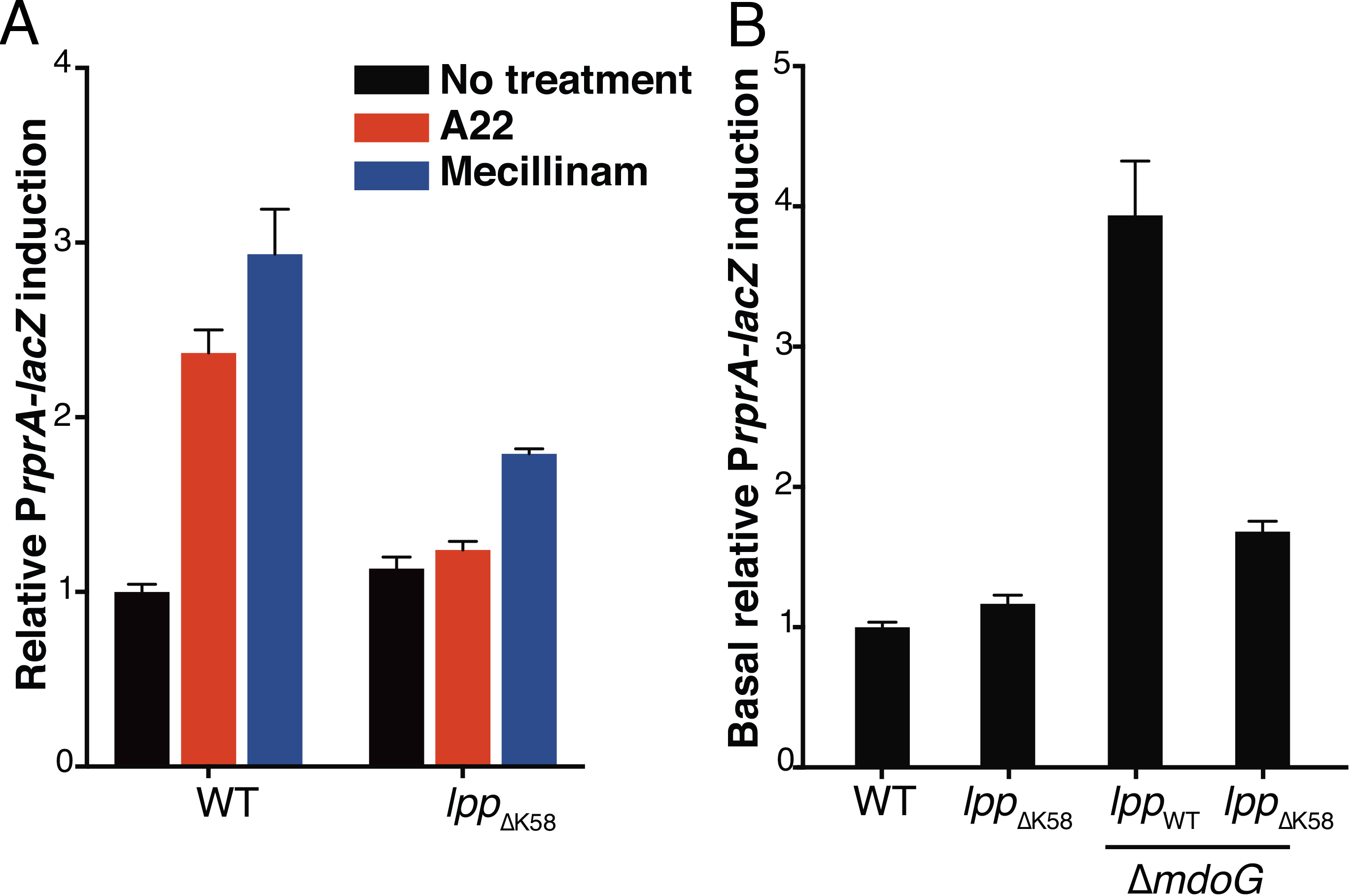

Supplement: S3 Fig — β-galactosidase activity was measured as in Fig 1. (A) Cells harbouring LppΔK58 (encoded on the chromosome at the lpp locus) were unable to activate the Rcs system in response to 5 μg/ml A22 or 0.3 μg/ml mecillinam, compared to WT (F(2, 27) = 14.66, P < 0.0001, 2-way ANOVA). (B) The lppΔK58 mutant also displayed an impaired Rcs response to mdoG deletion (F(3, 20) = 45.86, P < 0.0001, 1-way ANOVA). All values were normalised by the average activity for untreated WT cells. Error bars depict standard error of the mean (n = 6 for A22 and n = 3 for mecillinam). Lpp, Braun’s lipoprotein; Rcs, regulation of capsule synthesis; WT, wild-type. (TIF) [file pbio.2004303.s005.tif]

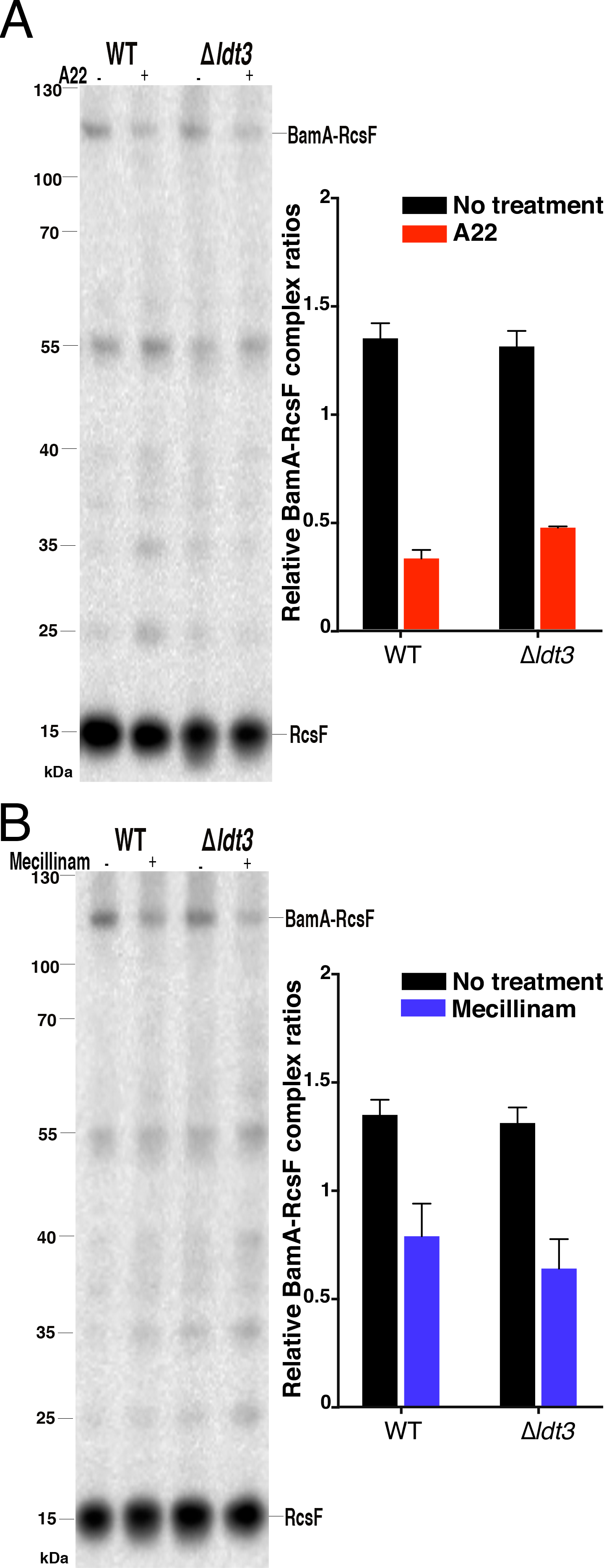

Supplement: S4 Fig — (A, B) Immunoblots show that levels of the BamA-RcsF complex were significantly lower after treatment (A) with A22 (5 μg/ml) or (B) with mecillinam (0.3 μg/ml) in WT and mutant strains (F(2, 12) = 50.48, P < 0.0001, 2-way ANOVA). BamA-RcsF complex levels were normalised to unspecific cross-reacting bands of the RcsF antibody. BamA-RcsF ratios were calculated relative to their levels in the no-stress condition for each strain. Representative data are shown from experiments performed in biological triplicate. Lpp, Braun’s lipoprotein; WT, wild-type. (TIF) [file pbio.2004303.s006.tif]

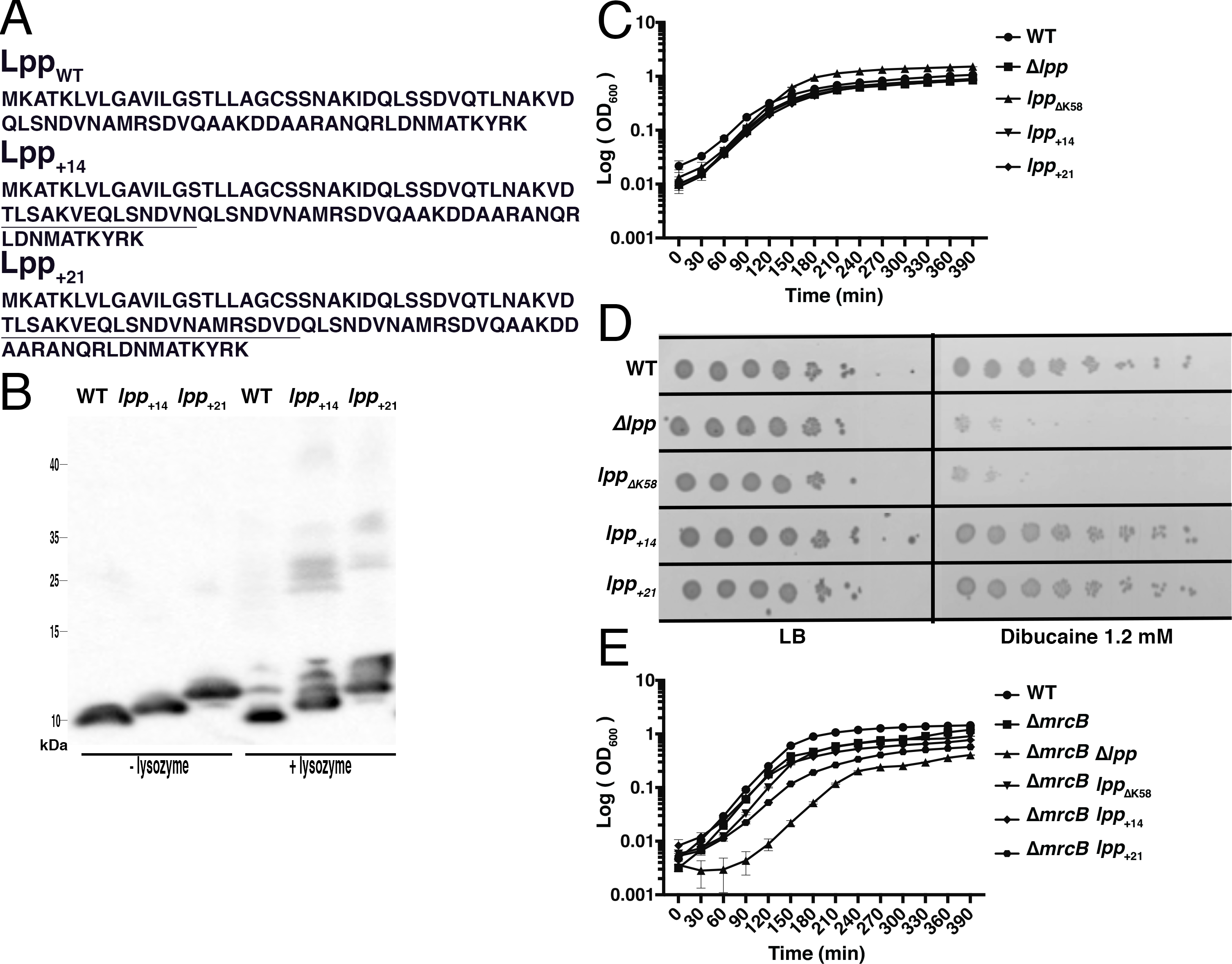

Supplement: S5 Fig — (A) Linear sequences of lpp mutants with insertions of 14 or 21 residues (2 and 3 heptad repeats, respectively). Insertions are underlined. (B) All Lpp variants expressed at similar levels from the chromosome cross-link to peptidoglycan. Lpp-peptidoglycan cross-links are detected in samples treated with lysozyme. Representative data are shown from experiments performed in biological triplicate. (C) Cells expressing Lpp+14 or Lpp+21 from the chromosome grow similarly to WT and to Δlpp and lppΔK58 mutants at 37°C. Values are averaged from independent clones (n = 6); error bars depict standard deviation. (D) Cells expressing Lpp+14 or Lpp+21 from the chromosome are as resistant as WT to dibucaine, unlike Δlpp and lppΔK58 mutants. Representative data are shown from experiments performed in biological triplicate. (E) Cells bearing lpp+14 or lpp+21 on the chromosome in addition to the mrcB deletion exhibited no significant growth defect compared to ΔlppΔmrcB mutants. Growth analysis was performed (n = 6) and depicted as in panel (C). Lpp, Braun’s lipoprotein; WT, wild-type. (TIF) [file pbio.2004303.s007.tif]

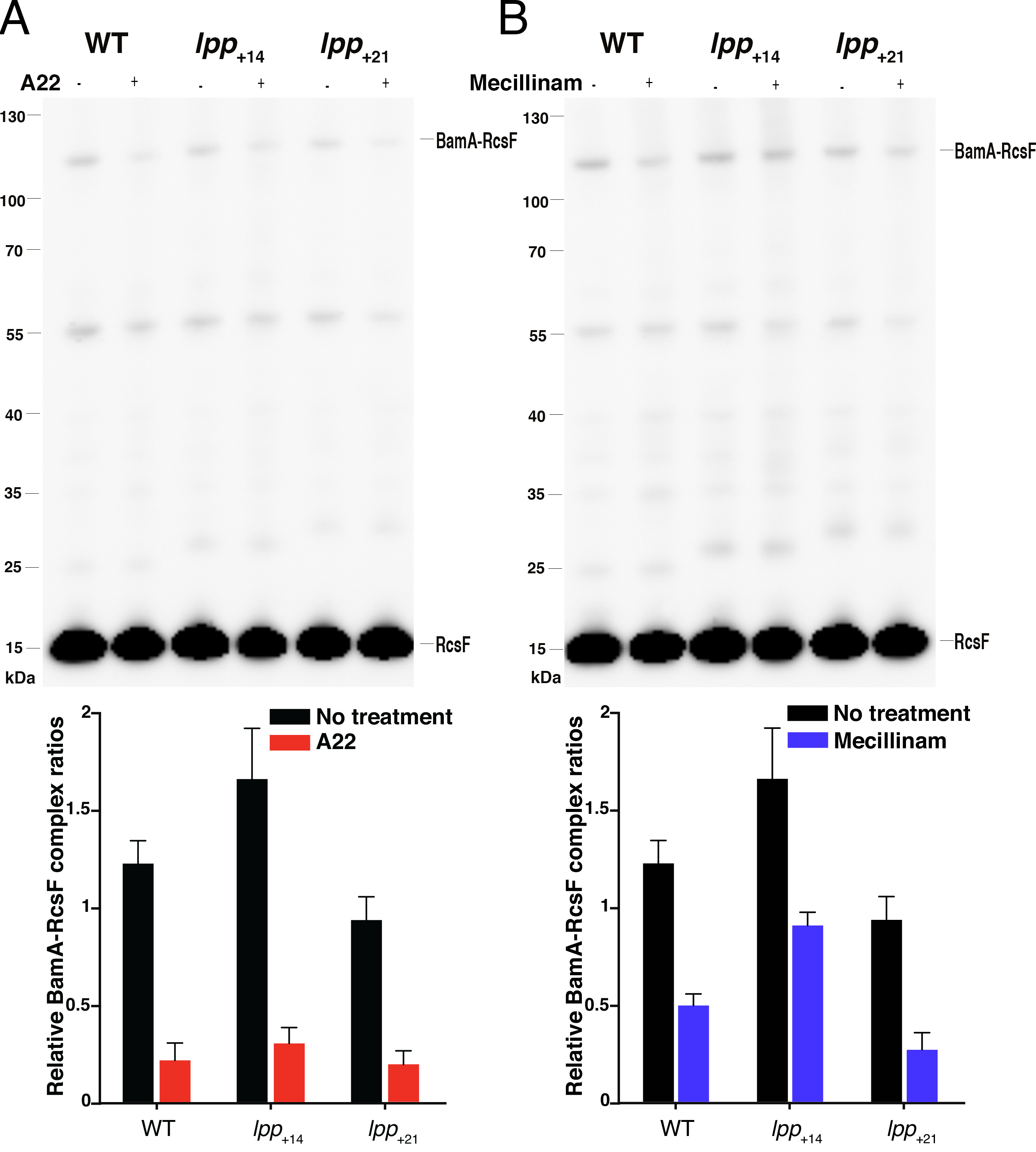

Supplement: S6 Fig — (A, B) Immunoblots indicate that levels of the BamA-RcsF complex were significantly lower after treatment (A) with 5 μg/ml A22 or (B) with 0.3 μg/ml mecillinam in WT and mutant strains (F(2, 18) = 58.08, P < 0.0001, 2-way ANOVA). Cells were treated with (A) A22 or (B) mecillinam and harvested after 40 min or 1 h, respectively. DTSSP cross-linking was performed, followed by SDS-PAGE and immunoblotting with an anti-RcsF antibody, as in S4 Fig. BamA-RcsF complex levels were normalised to unspecific cross-reacting bands of the RcsF antibody. BamA-RcsF ratios were calculated relative to their levels in the no-stress condition for each strain. Representative data are shown from experiments performed in biological triplicate. DTSSP, 3,3′-dithiobis[sulfosuccinimidylpropionate]; Lpp, Braun’s lipoprotein; SDS-PAGE, Sodium dodecyl sulfate–polyacrylamide gel electrophoresis; WT, wild-type. (TIF) [file pbio.2004303.s008.tif]

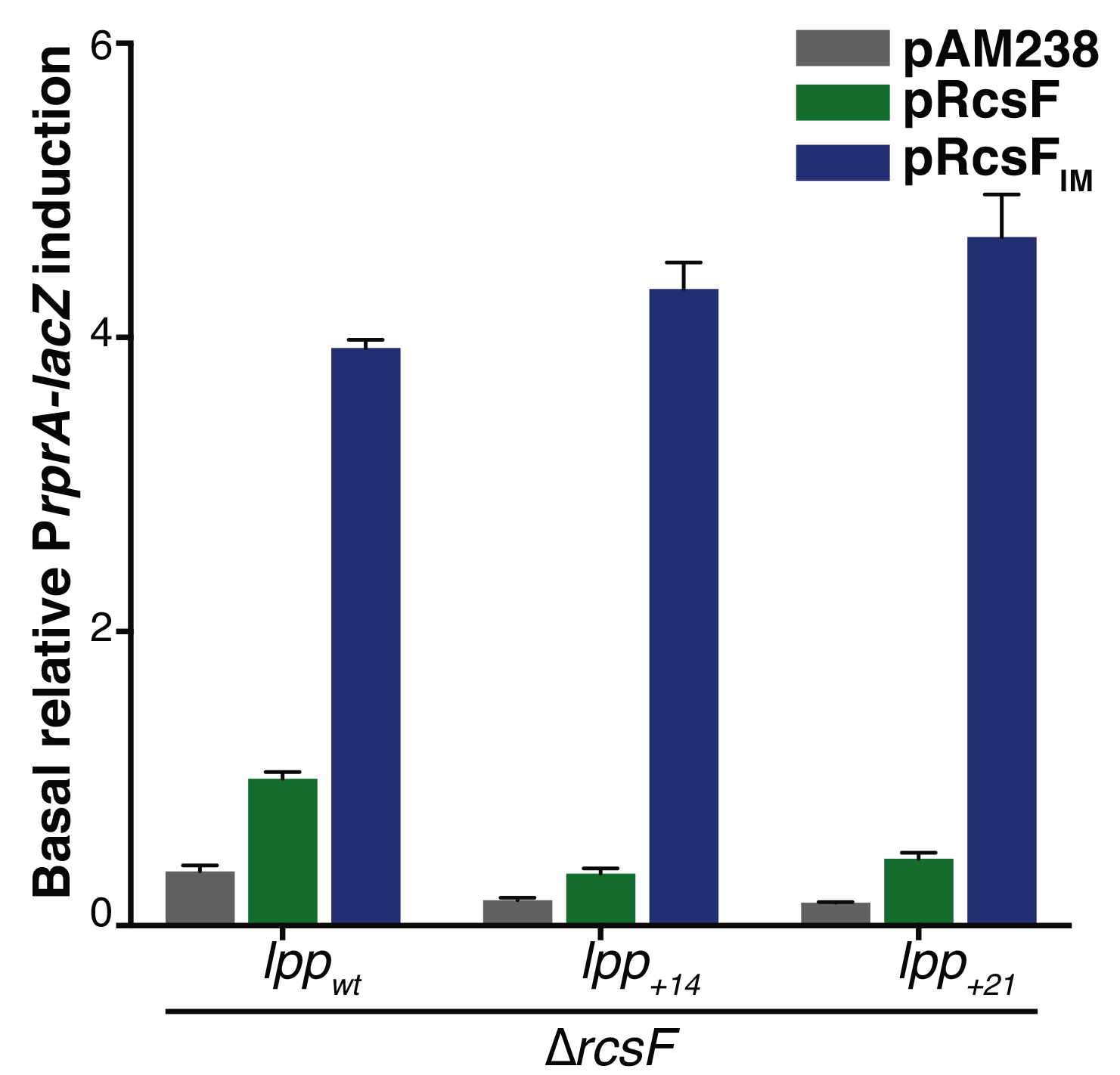

Supplement: S7 Fig — β-galactosidase activity was measured as in Fig 1. Expressing the RcsF mutant retargeted to the IM (RcsFIM) from a low-copy plasmid (pAM238) constitutively activated the Rcs response in cells expressing Lpp+14 and Lpp+21, as in cells expressing LppWT. Activation levels were significantly higher when expressing RcsFIM (F(16, 43) = 187.2, P < 0.0001, 1-way ANOVA). All values were normalised to the average activity obtained for untreated ΔrcsF cells, expressing lppWT and harbouring pRcsF. Error bars represent standard error of the mean (n = 3). IM, inner membrane; Lpp, Braun’s lipoprotein; Rcs, regulation of capsule synthesis. (TIF) [file pbio.2004303.s009.tif]

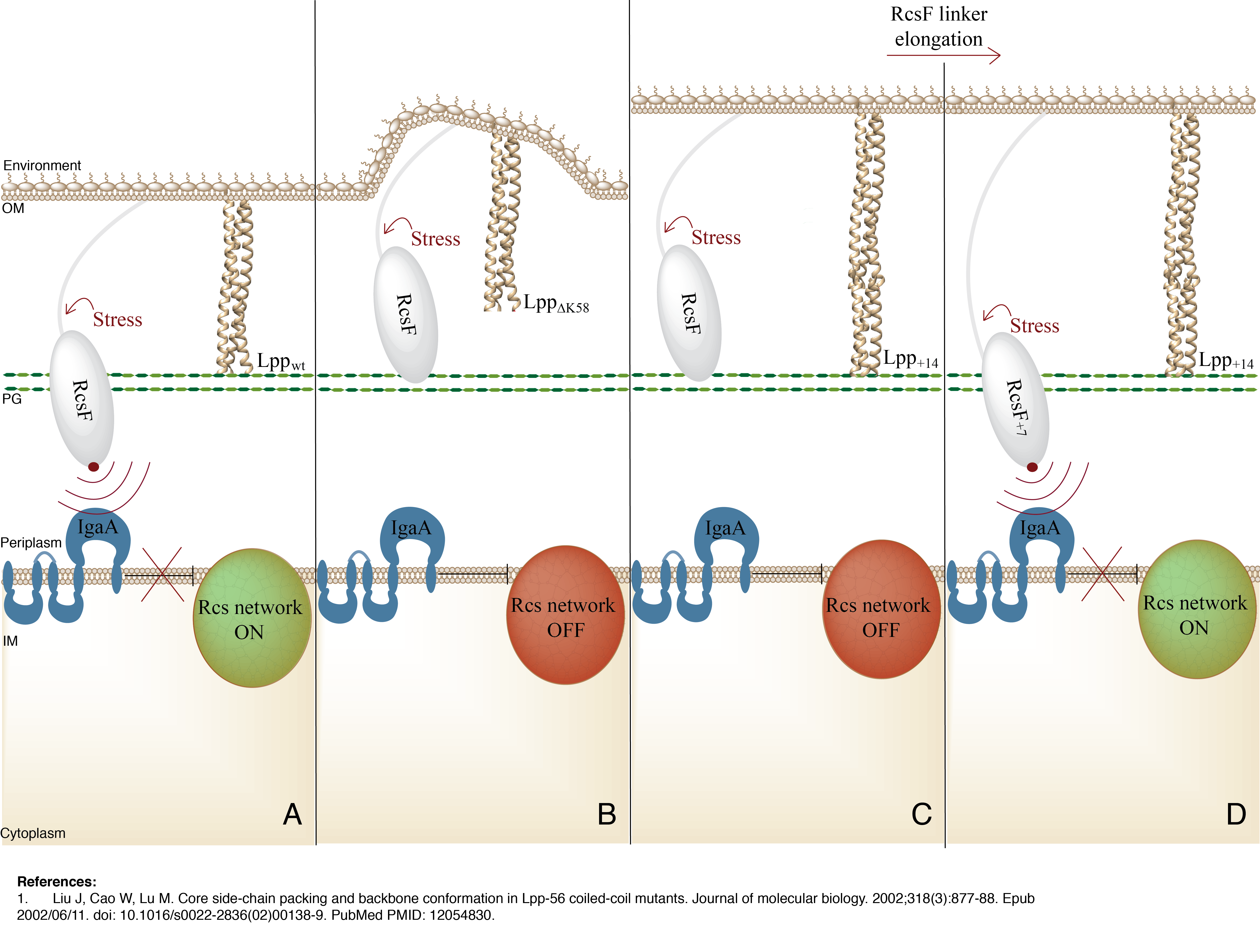

Supplement: S9 Fig — When the bacterium is subjected to stress in the OM or in the peptidoglycan, it activates the Rcs system to control and fix the damage. (A) In order to activate Rcs, the stress sensor lipoprotein RcsF, which is localised in the OM, must cross the periplasm to reach the IM protein IgaA. IgaA functions as the down-regulator of the Rcs system. When interacting with RcsF, IgaA alleviates its inhibition of Rcs, triggering the Rcs response. The architecture of the cell envelope needs to be tightly controlled to allow the information to be transmitted from RcsF in the OM to IgaA in the IM. In WT cells, the IM-to-OM distance is maintained by Lpp, a trimeric [46] lipoprotein that cross-links the OM to the peptidoglycan. (B) Preventing the attachment of Lpp to the peptidoglycan (LppΔK58) or (C) making this lipoprotein longer (Lpp+14 and Lpp+21) increases the width of the periplasm, which disrupts the line of communication between the 2 membranes. (D) Increasing the length of the RcsF N-terminal linker restores Rcs signalling by allowing RcsF to span the wider cell envelope and contact IgaA. IM, inner membrane; Lpp, Braun’s lipoprotein; OM, outer membrane; Rcs, regulation of capsule synthesis; WT, wild-type. (TIF) [file pbio.2004303.s011.tif]
