# Supplementary material for: Communication across the bacterial cell envelope depends on the size of the periplasm
Source: PLoS Biol. 2017 Dec 19;15(12):e2004303. doi: 10.1371/journal.pbio.2004303 (PMC5736177; doi:10.1371/journal.pbio.2004303)
Supplement: S8 Fig — (A) The functional domains of RcsF. The signal sequence is followed by the lipobox, which contains the acylated cysteine, the first amino acid of the mature lipoprotein. Amino acids at positions +2 and +3 after the cysteine form the Lol sorting signal that allows RcsF to be exported to the outer membrane. The globular signalling domain is preceded by an unstructured linker of 32 residues. (B) Primary sequences of RcsFWT and RcsF+7. Colours correspond to the colours in (A). The linker region (yellow) is rich in disorder-promoting amino acids (proline, alanine, lysine, arginine). To construct RcsF+7, 7 disordered residues from the WT linker sequence were duplicated. The duplicated peptide in RcsF+7 is underlined in yellow. (C) Ribbon representation of the RcsFWT structure (PDB: 2L8Y). This structure, which was solved via nuclear magnetic resonance, contains a flexible linker in the N-terminal part of RcsFWT [31]. The duplicated residues from RcsFWT used to construct RcsF+7 are highlighted in yellow on the ribbon using UCSF Chimera [43]. Peptide length was predicted by PyMOL [44] as 2–3 nm. (D) PrDOS [45] prediction of disordered regions in RcsFWT and RcsF+7. RcsF+7 shows disorder in its extended linker that is similar to that in the WT linker. The input sequence starts at the acylated cysteine residue. The sequence is predicted to be disordered when the disorder probability/confidence score is >0.5. The orange line shows the confidence of disordered protein-binding residue predictions. False positive rates = 5%. Lol, lipoprotein outer membrane localization; PrDOS, protein disorder prediction server; WT, wild-type. (DOCX) [file pbio.2004303.s010.docx]

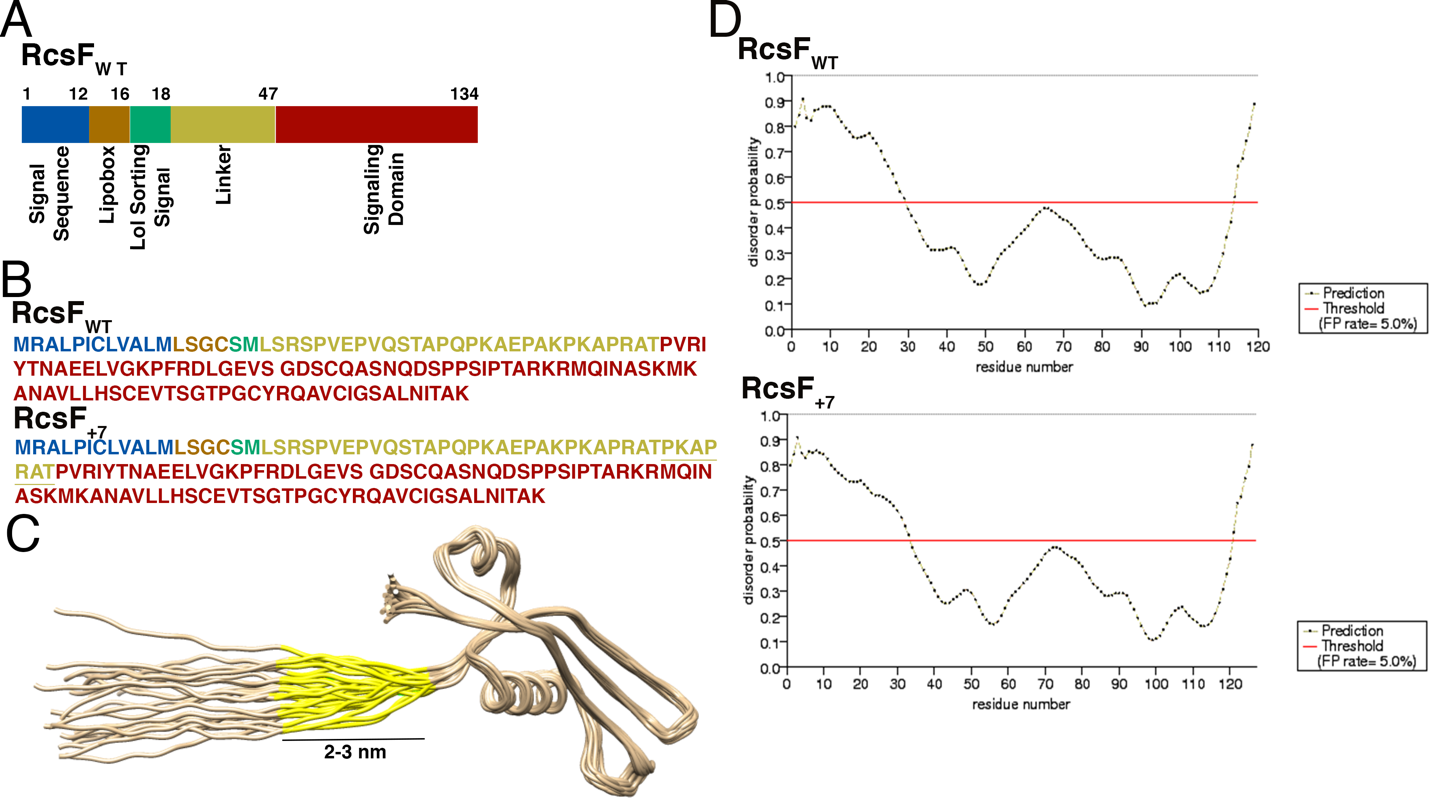


(A) The functional domains of RcsF. The signal sequence is followed by the lipobox, which contains the acylated cysteine, the first amino acid of the mature lipoprotein. Amino acids at positions +2 and +3 after the cysteine form the Lol sorting signal that allows RcsF to be exported to the outer membrane. The globular signalling domain is preceded by an unstructured linker of 32 residues. (B) Primary sequences of RcsF_WT_ and RcsF_+7_. Colours correspond to the colours in (A). The linker region (yellow) is rich in disorder-promoting amino acids (proline, alanine, lysine, arginine). To construct RcsF_+7_, seven disordered residues from the WT linker sequence were duplicated. The duplicated peptide in RcsF_+7_ is underlined in yellow. (C) Ribbon representation of the RcsF_WT_ structure (PDB: 2L8Y). This structure, which was solved via nuclear magnetic resonance, contains a flexible linker in the N-terminal part of RcsF_WT_[1]. The duplicated residues from RcsF_WT_ used to construct RcsF_+7_ are highlighted in yellow on the ribbon using UCSF Chimera[2]. Peptide length was predicted by PyMOL[3] as 2-3 nm. (D) PrDOS[4] prediction of disordered regions in RcsF_WT_ and RcsF_+7_. RcsF_+7_ shows disorder in its extended linker that is similar to that in the WT linker. The input sequence starts at the acylated cysteine residue. The sequence is predicted to be disordered when the disorder probability/confidence score is > 0.5. The orange line shows the confidence of disordered protein-binding residue predictions. False positive rates = 5%.

**References:**

1. Rogov VV, Rogova NY, Bernhard F, Löhr F, Dötsch V. A Disulfide Bridge Network within the Soluble Periplasmic Domain Determines Structure and Function of the Outer Membrane Protein RCSF. The Journal of Biological Chemistry. 2011;286(21):18775-83. doi: 10.1074/jbc.M111.230185. PubMed PMID: PMC3099694.

2. Pettersen EF, Goddard, T.D., Huang, C.C., Couch, G.S., Greenblatt, D.M., Meng, E.C., and Ferrin, T.E. . "UCSF Chimera - A Visualization System for Exploratory Research and Analysis." J Comput Chem 2004;25:1605-12

3. Delano WL. The PyMOL Molecular Graphics System. 2002. doi: citeulike-article-id:2816763.

4. Ishida T, Kinoshita K. PrDOS: prediction of disordered protein regions from amino acid sequence. Nucleic acids research. 2007;35(Web Server issue):W460-4. Epub 2007/06/15. doi: 10.1093/nar/gkm363. PubMed PMID: 17567614; PubMed Central PMCID: PMCPMC1933209.
